# Effectiveness of modified clear aligner designs in mesialization of permanent molars: a typodont model study

**DOI:** 10.3389/fdmed.2025.1645821

**Published:** 2025-10-13

**Authors:** Sara Mohamed Al Ali, Ahmed Ghoneima

**Affiliations:** ^1^ Department of Orthodontics and Pediatric Dentistry, Hamdan Bin Mohammed College of Dental Medicine (HBMCDM), Mohammed Bin Rashid University of Medicine and Health Sciences (MBRU), Dubai, United Arab Emirates; ^2^ Section of Orthodontics, School of Dentistry, University of California, Los Angeles (UCLA), Los Angeles, CA, United States

**Keywords:** clear aligners, molar mesialization, shape memory polymer, orthodontic biomechanics, attachment design, tipping vs. bodily movement

## Abstract

**Introduction:**

Clear aligner therapy has evolved significantly as an aesthetic and comfortable alternative to conventional fixed orthodontic appliances. However, the predictability of certain complex tooth movements such as bodily mesialization of molars remains a subject of ongoing investigation. This study explored the efficacy of clear aligners made from shape memory polymer (Graphy Tera Harz TC-85DAC resin) in achieving mesialization of the upper first molar.

**Methods:**

This *in vitro* study was conducted using typodont models with an extracted upper second premolar. Digitally designed clear aligners were fabricated using shape memory polymer. Four groups were assessed based on attachment design: no attachments, buccal attachment, palatal attachment, and both buccal and palatal attachments. The aligners were designed to move the upper first molar mesially by 3 mm. Pre- and post-treatment positions of the molars were measured and analyzed to determine the nature and extent of tooth movement.

**Results:**

All groups demonstrated mesial movement of the upper left first molar; however, only Groups 1 (no attachments) and 2 (buccal and palatal attachments) achieved the planned 3 mm movement. Groups 3 and 4 exhibited slightly less movement. The distance between the upper first molar and upper first premolar reduced significantly in all groups (*P* < 0.001). All groups showed mesial tipping rather than bodily movement of the molar. No attachment configuration was able to produce controlled root movement into the space.

**Conclusion:**

The modified clear aligner design fabricated from shape memory polymer (Graphy material) induced mesial tipping of the upper first molar but failed to achieve bodily mesialization. Further research is necessary to optimize aligner design and biomechanical strategies to enable more predictable control in mesial molar movement.

## Introduction

Despite the longstanding status of conventional fixed orthodontic appliances (brackets and archwires) as the gold standard for managing a wide spectrum of malocclusions, they are not without limitations. Patients frequently report challenges such as maintaining optimal oral hygiene, discomfort due to mucosal irritation, dental pain, and dissatisfaction with their aesthetic appearance. In response to these concerns, alternative orthodontic modalities have emerged, including ceramic brackets, lingually placed appliances, and clear aligners, aiming to provide improved patient comfort and enhanced aesthetics without compromising treatment outcomes (1–3).

Clear aligners are a series of customized, transparent, removable thermoplastic trays designed to incrementally move teeth into optimal positions. Each aligner is typically worn for one to two weeks, for a minimum of 20 h per day, to achieve the desired tooth movement. In cases of mild to moderate malocclusion, clear aligners have demonstrated advantages over conventional fixed appliances, including shorter treatment duration, reduced chair time, and greater cost-effectiveness, making them a viable and efficient alternative. The clear aligner technique relies heavily on digital technologies for treatment planning and appliance fabrication. A complete set of sequenced aligners is designed and manufactured digitally, tailored to the specific orthodontic needs of each patient. The integration of a digital workflow including intraoral scanning, computer-aided design (CAD), and computer-aided manufacturing (CAM), and three-dimensional (3D) printing has significantly enhanced the precision, predictability, and reproducibility of clear aligner therapy. These technological advancements enable clinicians to generate comprehensive, individualized treatment plans, while allowing patients to visualize simulated treatment progress and anticipated outcomes prior to initiation, thereby improving patient understanding and engagement (4–6).

A wide range of dental malocclusion cases can be treated using clear aligners, however; their effectiveness is limited with respect to certain types of tooth movements. Although it is often regarded superior to conventional fixed appliances in terms of aesthetics, comfort, and patient acceptance, significant gaps remain in the literature concerning the predictability of specific orthodontic tooth movements achieved through this modality. Complex orthodontic tooth movements are difficult to achieve using clear aligners alone without adjuncts. Consequently, the success of clear aligner therapy is highly contingent upon the clinician's understanding of orthodontic biomechanics. One of the critical factors in optimizing treatment outcomes is effective anchorage control, which plays a significant role in enhancing the predictability and precision of tooth movement. An in-depth knowledge of force systems, aligner limitations, and strategic use of attachments or auxiliary devices is essential for achieving desired clinical results in complex cases (7–9). The aim of the current study was to evaluate the effectiveness of a modified clear aligner design in achieving mesialization of permanent molars by assessing the type of tooth movement generated (tipping vs. bodily movement) and comparing outcomes with and without the use of attachments.

## Materials and methods

This *in vitro* study was conducted on a total of 400 clear aligners divided into four groups (*n* = 100/group) based on the attachment location. Group 1 included aligners without attachments, Group 2 included aligners with attachments placed on both buccal and palatal surfaces, Group 3 included aligners with attachments placed on the palatal surface only, and Group 4 included aligners with attachments placed on the buccal surface only. The sample size was sufficient to detect the observed tooth movement with a power greater than 80% at an α-level of 0.05.

**Figure 1:**
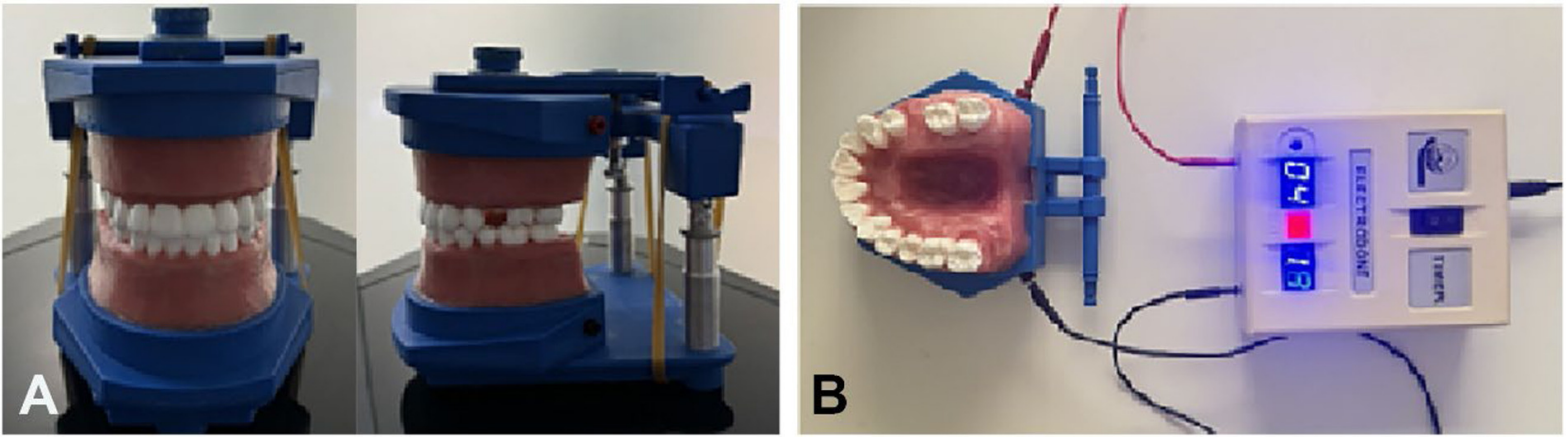
**(A)** The typodont (electrodont) model used in the study, **(B)** the electrodont model attached to power circuit.

An electric typodont model (Electro-Dont; Savaria-Dent, Budapest, Hungary), designed to simulate tooth movement, was used in the study. The maxillary left second premolar was removed from the typodont to create space for testing mesial movement of the maxillary left first permanent molar (Figure 1). The typodont was scanned using the RAYIOS2 intraoral scanner (DDS Comfort+, Seoul, Korea) to generate a digital replica. The resulting digital models were saved in stereolithography (STL) file format and uploaded into Maestro 3D Ortho Studio® software (AGE Solutions®, Pontedera, Italy) for tooth segmentation and virtual tooth movement planning. Tooth segmentation was performed by outlining the cervical margin of each tooth. The Maestro software includes a built-in virtual tooth movement module that simulates tooth displacement in three planes of space, allowing alignment with the planned movement. A standardized mesial movement of 3 mm was applied to the maxillary left first permanent molar in all models.

**Figure 2:**
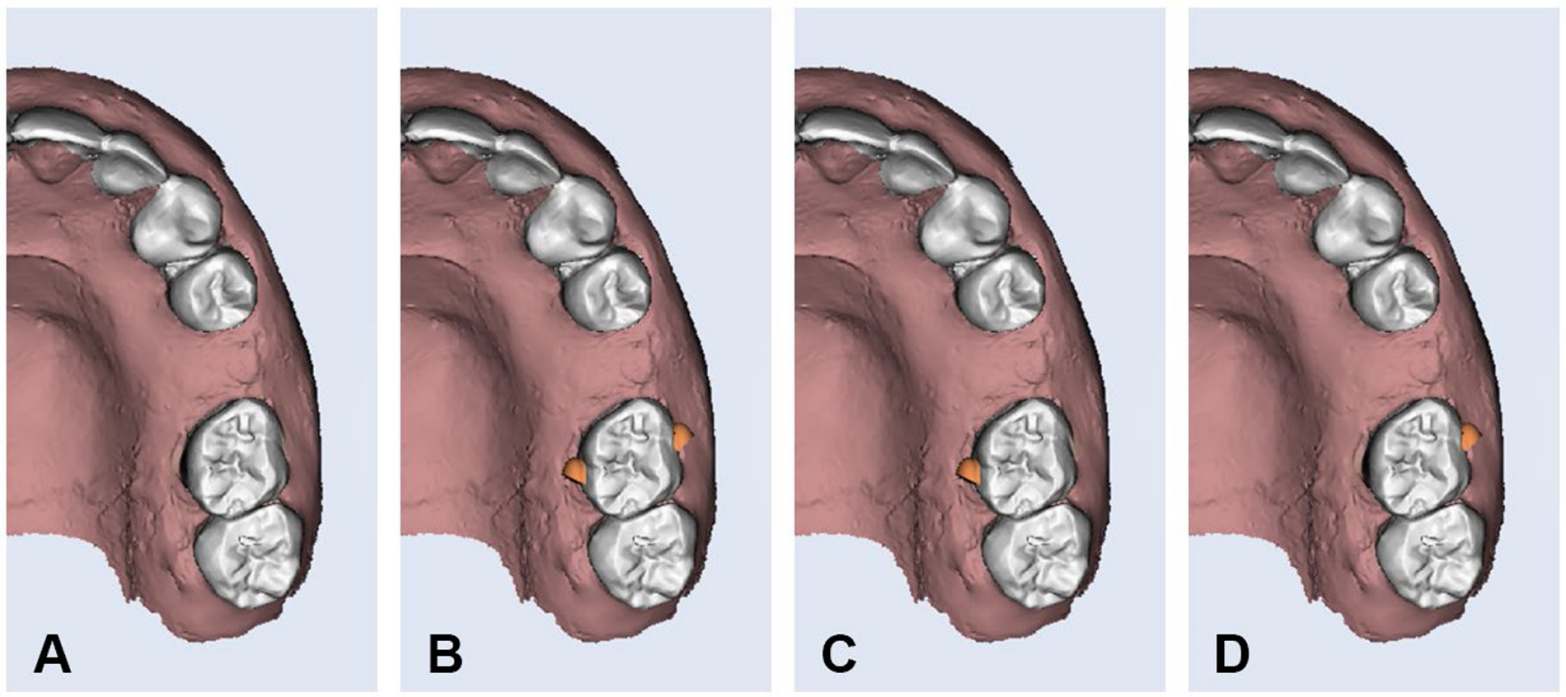
Optimized attachment design on buccal and palatal surfaces of upper left first molar. **(A)** Group-1, **(B)** Group-2, **(C)** Group-3, and **(D)** Group-4.

Aligners were manufactured with a thickness of 0.5 mm using Graphy Tera Harz TC-85DAC resin (Graphy Inc, Seoul, Korea), a shape memory polymer material that is designed for the production of clear aligners. Optimized attachments were designed with precise dimensions of 4 mm in width, 3 mm in height, and 2 mm in depth for all aligners in Groups 2, 3, and 4. A distal bevel was added to the design to facilitate the mesialization movement. Attachments were placed using the same attachment template in all groups (Figure 2). The attachment aligner interface remained intact in all specimens. Ten progressive aligners (Aligners 1–10) were digitally designed using standardized procedures. Each aligner was programmed to achieve an incremental mesial movement of 0.3 mm, resulting in a total of 3 mm of movement by the completion of Aligner 10. A baseline aligner (Aligner 0) was designed to establish and standardize the initial position of the maxillary left first molar at the beginning of each cycle.

**Figure 3:**
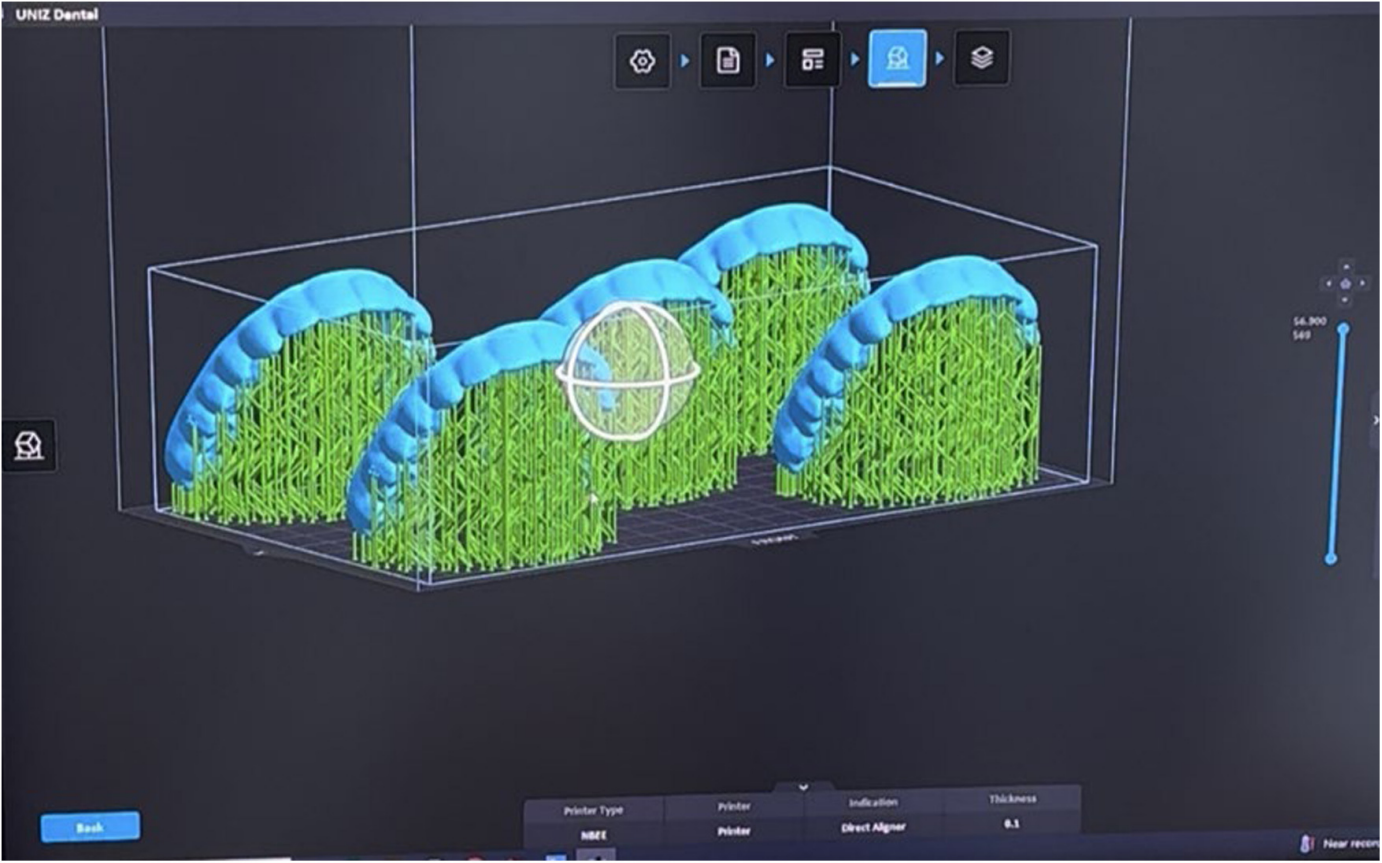
Aligners positioned vertically with an angulation of 20° to the printing platform.

The aligners were printed using a Uniz Slash-C LCD 3D printer (Uniz, San Diego, CA, USA). They were positioned vertically at a 20° angle to the build platform and printed simultaneously (Figure 3). After printing, the aligners were detached from the platform using a detaching device (UNIZ U Detach), then placed in a Tera Harz Spinner (THS-heater centrifuge) for 6 min to remove excess resin through centrifugal spinning. The final step involved curing the aligners in a nitrogen curing chamber (Tera Harz Cure) for 20 min to complete the polymerization process.

To evaluate maxillary molar mesialization, tooth movement was tested ten times in each group. Within each cycle, aligners were sequentially numbered from 1 to 10, with each set representing one complete cycle of simulated tooth movement. The ElectroDont device was used to deliver a controlled thermal cycle, consisting of a 10 min heating phase to gradually soften the wax, followed by a 10 min cooling phase for solidification. In accordance with the manufacturer's instructions, the ElectroDont was connected to a power source after positioning aligner-1 over the dental model.

The aligner remained in place during the 10 min heating period, during which the generated heat progressively melted the wax, allowing the programmed tooth movement to occur. This was followed by a 10 min cooling phase with the aligner still in position to enable wax solidification. To ensure complete hardening of the wax, the model was then immersed in room-temperature water for an additional 5 min before applying the next aligner. This entire process was repeated systematically for each aligner (Aligners 1–10) across all experimental groups. Upon completing each cycle of aligners, the model was reheated, and the teeth were reset to their initial positions using aligner-0 before initiating the next cycle.

**Figure 4:**
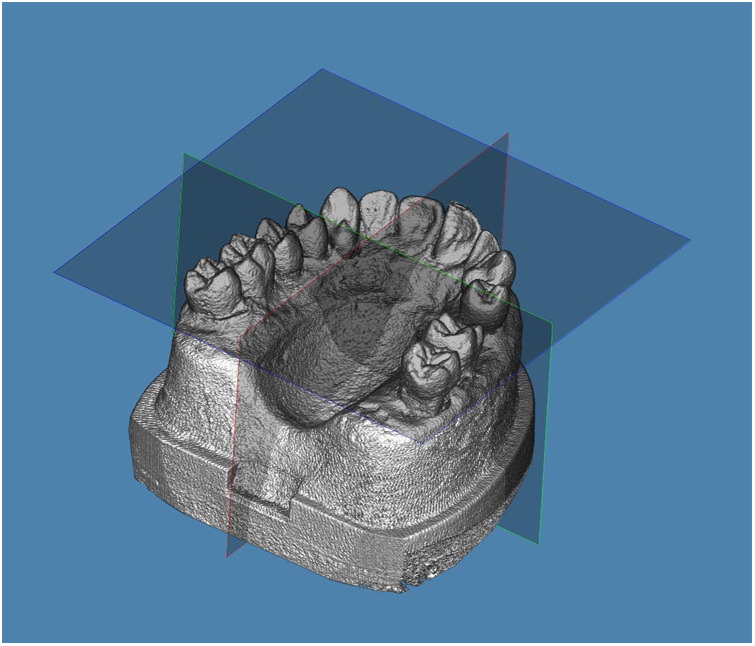
Models were oriented with the midsagittal plane passing through the midline, axial plane representing the occlusal plane by touching the cusp tips of the last molars and canines, and transverse plane passing through the mesial aspect of the maxillary second molars and perpendicular to the occlusal plane.

Cone-beam computed tomography (CBCT) scans were taken at baseline (Aligner 1) and after completing the full tooth movement (Aligner 10), using the Veraviewepocs 3D R100 system (J. Morita Mfg. Corp., Kyoto, Japan). For consistent orientation, the midsagittal plane was aligned with the midline of the model, the axial plane represented the occlusal plane by contacting the cusp tips of the last molars and canines, and the transverse plane passed through the mesial aspect of the maxillary second molars, perpendicular to the occlusal plane (Figure 4).

**Figure 5:**
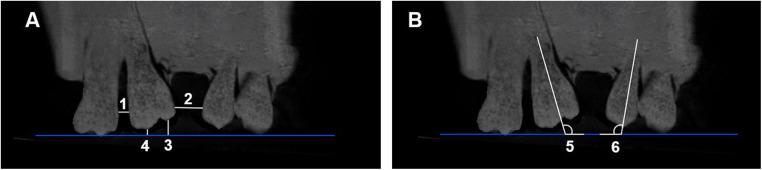
**(A)** The linear and **(B)** angular measurements selected in the study. (1) UL7—UL6 (mm), (2) UL6—UL4 (mm), (3) UL6 MC—OP (mm), (4) UL6 DC—OP (mm), (5) UL6—OP°, and 6) UL4—OP°.

Four linear and two angular measurements (Table 1 and Figure 5) were performed using Dolphin 3D software (Dolphin Imaging 11.0, Dolphin Imaging and Management Solutions, Chatsworth, CA) to assess the type and extent of tooth movement in each group. To ensure methodological reliability, the full experimental protocol was repeated ten times per group.

**Table 1:** Description of measurements used in the study.

Measurement	Definition
UL7—UL6 (mm)	Horizontal linear distance between mesial contact point of upper left second molar and distal contact point of upper left first molar
UL6—UL4 (mm)	Horizontal linear distance between mesial contact point of upper left first molar and distal contact point of upper left first premolar
UL6 MC—OP (mm)	Vertical linear distance between occlusal plane and mesial cusp tip of upper first molar (occlusal plane drawn occlusally from upper left second molar to upper left central incisor)
UL6 DC—OP (mm)	Vertical linear distance between occlusal plane and distal cusp tip of upper first molar (occlusal plane drawn occlusally from upper left second molar to upper left central incisor)
UL6—OP (angle)	Angle between occlusal plane and long axis of upper left first molar
UL4—OP (angle)	Angle between occlusal horizontal plane and long axis of upper left first premolar

MC, mesial cusp; OP, occlusal plane; DC, distal cusp.

Prior to data collection, the primary investigator (S.A.) assessed intra-rater reliability by measuring the selected parameters on two separate occasions, two weeks apart, using a random sample of 10 CBCT scans.

### Statistical analysis

Statistical analyses were conducted using SPSS software version 29.0 (SPSS Inc., Chicago, IL, USA). Continuous variables were summarized using means, standard deviations, minimum, and maximum values for each group. The normality of the measurements was assessed using the Shapiro–Wilk test. Based on the distribution of data, paired *t*-tests were used to compare pre- and post-movement values within each group, while independent *t*-tests were applied to compare means between two independent groups. A *P*-value of less than 0.05 was considered statistically significant.

## Results

Intra-rater reliability was excellent as indicated by intraclass correlation coefficients (ICCs) ≥ 0.90. No statistically significant differences were observed between the two measurements.

Comparisons of pre- and post-tooth movement within each group are summarized in Tables 2–5. Table 2 presents the measurements before and after tooth movement in Group 1. The mean horizontal distances between the upper left second molar (UL7) and first molar (UL6), and between the upper left first molar (UL6) and first premolar (UL4), at the beginning of the cycle were 0.57 mm and 9.7 mm, respectively. Following the completion of the tooth movement cycle, notable changes were observed. The UL6 moved mesially by 2.31 mm, and the distance between UL6 and UL4 decreased by 2.7 mm, indicating significant mesial movement of the first molar. Regarding vertical measurements, the distances between the mesial and distal cusp tips of UL6 were initially 1.06 mm and 0.7 mm, respectively. This indicated that the mesial cusp tip was at a lower vertical level than the distal cusp tip before movement. After tooth movement, both distances increased, reflecting vertical positional changes of the cusps during mesialization. In terms of angular measurements, the initial angle between UL6 and the occlusal plane was 90.7°, while that between UL4 and the occlusal plane was 92°, suggesting a slight mesial tipping of UL4 relative to UL6. By the end of the cycle, the tipping of UL4 increased by 2.6°, whereas UL6 exhibited a more pronounced increase in mesial tipping, with a change of 9.2° from baseline. All changes observed in Group 1 were statistically significant.

**Table 2:** Comparison of pre- and post-tooth movement measurements in group 1 (no attachments).

Measurement	T1		T2		Difference		95% CI		*P* value
	Mean	SD	Mean	SD	Mean	SD	Lower	Upper	
UL7—UL6 (mm)	0.57	0.29	2.88	0.44	2.31	0.59	−2.73	−1.89	0.001*
UL6—UL4 (mm)	9.70	0.71	6.97	0.43	−2.73	0.68	2.25	3.21	0.001*
UL6 MC—OP (mm)	1.06	0.38	2.27	0.86	1.21	0.97	−1.90	−0.52	0.003*
UL6 DC—OP (mm)	0.70	0.28	1.47	0.45	0.77	0.46	−1.10	−0.45	0.001*
UL6—OP (angle)	90.71	1.31	99.97	2.14	9.26	1.79	−10.54	−7.98	0.001*
UL4—OP (angle)	92.10	1.83	94.69	2.36	2.59	2.35	−4.27	−0.91	0.007*

* Significant at ≤0.05.

**Table 3:** Comparison of pre- and post-tooth movement measurements in group 2 (attachments on both buccal and palatal surfaces).

Measurement	T1		T2		Difference		95% CI		*P* value
	Mean	SD	Mean	SD	Mean	SD	Lower	Upper	
UL7—UL6 (mm)	0.52	0.18	2.53	0.49	2.01	0.47	−2.34	−1.68	0.001*
UL6—UL4 (mm)	10.34	0.28	8.23	0.64	−2.11	0.61	1.67	2.55	0.001*
UL6 MC—OP (mm)	0.69	0.20	1.95	0.45	1.26	0.48	−1.60	−0.92	0.001*
UL6 DC—OP (mm)	0.54	0.17	1.31	0.33	0.77	0.38	−1.04	−0.50	0.001*
UL6—OP (angle)	90.81	1.07	96.35	2.68	5.54	2.18	−7.10	−3.98	0.001*
UL4—OP (angle)	91.08	0.84	92.71	1.60	1.63	1.40	−2.63	−0.63	0.005*

* Significant at ≤0.05.

**Table 4:** Comparison of pre- and post-tooth movement measurements in group 3 (attachments on the palatal surface only).

Measurement	T1		T2		Difference		*P* value
	Mean	SD	Mean	SD	Mean	SD	
UL7—UL6 (mm)	0.52	0.13	2.22	0.28	1.70	.27080	0.005*
UL6—UL4 (mm)	10.05	0.36	8.43	0.40	−1.62	.42635	0.005*
UL6 MC—OP (mm)	0.91	0.12	2.27	0.21	1.36	.20111	0.005*
UL6 DC—OP (mm)	0.58	0.23	1.28	0.20	0.70	.35901	0.005*
UL6—OP (angle)	91.40	0.60	97.43	0.59	6.03	.86929	0.005*
UL4—OP (angle)	91.43	0.45	96.00	0.67	4.57	.48774	0.005*

* Significant at ≤0.05.

**Table 5:** Comparison of pre- and post-tooth movement measurements in group 4 (attachments on the buccal surface only).

Measurement	T1		T2		Difference		95% CI		*P* value
	Mean	SD	Mean	SD	Mean	SD	Lower	Upper	
UL7—UL6 (mm)	0.38	0.09	1.90	0.28	1.52	0.28	−1.72	−1.32	0.001*
UL6—UL4 (mm)	9.92	0.33	7.72	0.30	−2.20	0.44	1.88	2.52	0.001*
UL6 MC—OP (mm)	0.95	0.14	2.55	0.41	1.60	0.43	−1.91	−1.29	0.001*
UL6 DC—OP (mm)	0.69	0.10	1.64	0.36	0.95	0.30	−1.17	−0.73	0.001*
UL6—OP (angle)	90.42	0.24	99.04	0.70	8.62	0.77	−9.17	−8.07	0.001*
UL4—OP (angle)	90.68	0.33	92.21	0.58	1.53	0.72	−2.05	−1.01	0.001*

* Significant at ≤0.05.

Table 3 presents the pre- and post-tooth movement values for Group 2 (attachments on both the buccal and palatal surfaces). The outcomes were similar to those observed in Group 1. Following the completion of tooth movement, the upper left first molar (UL6) shifted mesially by 2.0 mm, and the distance between UL6 and the upper left first premolar (UL4) decreased by 2.1 mm. In terms of vertical displacement, the distances from both the mesial and distal cusp tips of UL6 to the occlusal plane increased post-movement, indicating vertical changes in cusp position during mesialization. Regarding angular measurements, the initial angle between UL6 and the occlusal plane was 90.8°, while UL4 had an initial angle of 91°, suggesting minimal tipping at baseline. After movement, UL6 exhibited an increase in mesial tipping by 5.5°, and UL4 showed a tipping increase of 1.6°. All changes in linear and angular measurements in Group 2 were statistically significant.

Table 4 outlines the pre- and post-tooth movement values for Group 3 (palatal attachments only). Following tooth movement, the upper left first molar (UL6) shifted mesially by 1.7 mm, resulting in a corresponding reduction in the distance between UL6 and the upper left first premolar (UL4). Post-treatment, the vertical distances from both the mesial and distal cusp tips of UL6 to the occlusal plane increased, suggesting mesial tipping of the tooth rather than bodily movement. In terms of angular measurements, UL6 exhibited a tipping increase of 6.0°, while UL4 showed an increase of 4.57° after treatment. As with the previous groups, all linear and angular changes in Group 3 were statistically significant.

Table 5 presents the pre- and post-treatment values for Group 4 (buccal attachments only). After the tooth movement cycle, UL6 moved mesially by 1.5 mm, leading to a reduction in the distance between UL6 and UL4. Vertical measurements also showed an increase in the distances from the occlusal plane to both the mesial and distal cusp tips of UL6, consistent with mesial tipping. For angular measurements, the tipping of UL6 increased by 8.6°, while UL4 exhibited a minor increase of 1.5°. As observed in the previous groups, all changes in Group 4 were statistically significant.

### Comparative analysis across groups

Table 6 presents the comparison between Group 1 (no attachments) and Group 2 (buccal and palatal attachments). Among the six measurements analyzed, three showed statistically significant differences between the two groups: the distance between UL6 and UL4, the angle between UL6 and the occlusal plane, and the angle between UL4 and the occlusal plane. The remaining three measurements did not differ significantly.

**Table 6:** Comparison between group 1 and group 2 after tooth movement.

Measurement	Group 1		Group 2		Difference		95% CI		*P*-value
	Mean	SD	Mean	SD	Mean	SE	Lower	Upper	
UL7—UL6 (mm)	2.88	0.44	2.53	0.49	0.35	0.17	0.00	0.70	0.11
UL6—UL4 (mm)	6.97	0.43	8.23	0.64	−1.26	0.21	−1.68	−0.84	0.001*
UL6 MC—OP (mm)	2.27	0.86	1.95	0.45	0.32	0.24	−0.17	0.81	0.31
UL6 DC—OP (mm)	1.47	0.45	1.31	0.33	0.16	0.16	−0.16	0.48	0.38
UL6—OP (angle)	99.97	2.14	96.35	2.68	3.62	0.79	2.01	5.23	0.004*
UL4—OP (angle)	94.69	2.36	92.71	1.60	1.98	0.67	0.63	3.33	0.04*

* Significant at ≤0.05.

Table 7 presents the comparison between Group 1 and Group 3 (palatal attachment only). The significant differences in this comparison were limited to the distances between UL7–UL6, UL6–UL4, and the angle between UL6 and the occlusal plane. All other variables showed no statistically significant differences.

**Table 7:** Comparison group 1 and group 3 after tooth movement.

Measurement	Group 1		Group 3		Difference		95% CI		*P*-value
	Mean	SD	Mean	SD	Mean	SE	Lower	Upper	
UL7—UL6 (mm)	2.88	0.44	2.22	0.28	0.66	0.17	0.31	1.01	0.001*
UL6—UL4 (mm)	6.97	0.43	8.43	0.40	−1.46	0.21	−1.88	−1.04	0.001*
UL6 MC—OP (mm)	2.27	0.86	2.27	0.21	0.00	0.24	−0.49	0.49	1.0
UL6 DC—OP (mm)	1.47	0.45	1.28	0.20	0.19	0.16	−0.13	0.51	0.24
UL6—OP (angle)	99.97	2.14	97.43	0.59	2.54	0.79	0.93	4.15	0.00*
UL4—OP (angle)	94.69	2.36	96.00	0.67	−1.31	0.67	−2.66	0.04	0.11

* Significant at ≤0.05.

Table 8 compares Group 1 with Group 4 (buccal attachment only). Significant differences were recorded in the UL7–UL6 distance, UL6–UL4 distance, and the angle between UL4 and the occlusal plane. Other measurements did not differ significantly between the groups.

**Table 8:** Comparison between group 1 and group 4 after tooth movement.

Measurement	Group 1		Group 4		Difference		95% CI		*P*-value
	Mean	SD	Mean	SD	Mean	SE	Lower	Upper	
UL7—UL6 (mm)	2.88	0.44	1.90	0.28	0.98	0.17	0.63	1.33	0.001*
UL6—UL4 (mm)	6.97	0.43	7.72	0.30	−0.75	0.21	−1.17	−0.33	0.001*
UL6 MC—OP (mm)	2.27	0.86	2.55	0.41	−0.28	0.24	−1.17	−0.33	0.37
UL6 DC—OP (mm)	1.47	0.45	1.64	0.36	−0.17	0.16	−0.49	0.15	0.37
UL6—OP (angle)	99.97	2.14	99.04	0.70	0.93	0.79	−0.68	2.54	0.21
UL4—OP (angle)	94.69	2.36	92.21	0.58	2.48	0.67	1.13	3.83	0.01*

* Significant at ≤0.05.

The only variable that showed a statistically significant difference in all comparisons was the UL6–UL4 distance. The decrease in this distance was attributed to both the mesial movement of the upper left first molar and the distal tipping of the adjacent premolar into the extraction space.

## Discussion

Over the last two decades, clear aligner technology has evolved dramatically. As the thermoplastic material used in clear aligners deforms under pressure, the rebound force creates controlled orthodontic forces on teeth. While clear aligners were initially limited to the correction of mild Class I malocclusions characterised by minor spacing or crowding, the integration of various auxiliaries has significantly expanded their clinical applicability to more complex orthodontic cases (10–12). The aim of the current study was to evaluate the effectiveness of a modified clear aligner design in achieving mesialization of permanent molars by assessing the type of tooth movement generated (tipping vs. bodily movement) and comparing outcomes with and without the use of attachments.

The foundational work of Kesling (13), who emphasized the diagnostic and planning value of preliminary setups and thermoplastic tooth positioners, continues to influence contemporary aligner therapy. While it has been proposed that clear aligners primarily produce intrusive or tipping forces, with limited capacity for translational tooth movement, emerging evidence suggests otherwise. Elfouly et al. (14) reported that maxillary molar distalization of 2 mm did not result in significant molar tipping; however, buccal inclination and mesiobuccal rotation were observed during the distalization process. Simon et al. (15) demonstrated that bodily movements including premolar derotation, molar distalization, and incisor torque, can be effectively achieved with clear aligners, with upper molar distalization shown to be effective over distances of approximately 1.5 mm to 3 mm. However, mesialization remains a more complex and less predictable movement. According to Rossini et al. (5), molar mesialization using clear aligners is particularly challenging, often requiring higher forces and exhibiting lower predictability.

In this study, clear aligners were digitally designed to facilitate 3 mm of mesial movement of the upper left first molar into the space created by the extraction of the second premolar. The design did not intend to include any change in the angulation of the upper first molar nor the first premolar, however; the final position of the teeth in all groups indicated that there was mesial tipping of the upper left first molar and distal tipping of the upper left first premolar into the extraction space. Three attachment configurations (palatal only, buccal only, and both buccal and palatal) were tested on the first molar to assess whether attachment positioning influenced the nature and magnitude of tooth movement. No attachments were placed on the first premolar to counteract reactive tipping forces, which may have contributed to the observed distal inclination. These findings align with those of Baldwin et al. (16), who reported significant tipping of adjacent teeth during space closure following premolar extraction, and Drake et al. (17), who indicated that despite the intended programming for bodily protraction of the target tooth, the movement frequently resulted in uncontrolled tipping. In contrast, Simon et al. (15) reported a high level of accuracy (88%) in achieving bodily movement of the maxillary molars when a distalization of at least 1.5 mm was prescribed. The highest precision was observed when the movement was assisted by the application of attachments on the tooth surface. The discrepancy in mesial vs. distal tooth movements may be explained by the biomechanics of clear aligners, which rely on thermoplastic materials that generate push rather than pull forces. This mechanical limitation may favor bodily movement in the distal direction while predisposing mesial movements to tipping (18).

Lyu et al. (1) observed mesiolingual tipping of mandibular second molars during clear aligner therapy, which was mitigated through the use of modified lever arms (MLAs) that facilitated distal tipping and extrusion instead. These findings highlight the biomechanical limitations of clear aligners and the importance of incorporating auxiliaries to enhance force delivery and control. Despite growing interest in complex tooth movements with clear aligners, current literature still lacks sufficient evidence regarding the effectiveness and biomechanics of molar mesialization. According to the findings of this study, comparison of the upper left first molar position before and after movement revealed that Groups 1 (no attachments) and 2 (buccal and palatal attachments) achieved the planned 3 mm of mesialization as programmed in the aligner software. In contrast, Groups 3 (palatal attachment only) and 4 (buccal attachment only) exhibited less than 3 mm of mesial movement, despite identical treatment planning. All groups demonstrated mesial tipping of the upper left first molar into the extraction space, indicating that the observed movement was not purely translational. The consistency of this outcome across groups suggests a uniform response of the thermoplastic material used in the aligners.

Overall, the results demonstrated successful mesialization of the upper left first molar in all groups, regardless of the presence or location of attachments. However, the movement achieved was primarily tipping rather than bodily movement. This conclusion is supported by the consistent increase in the angle between UL6 and the occlusal plane across all four groups.

This study serves as a preliminary *in vitro* investigation into the use of thermally responsive shape memory polymers (SMPs) for orthodontic aligners. Tooth movement was successfully achieved on a typodont model through the shape recovery forces activated by thermal stimuli. The material used was selected for its flexibility, durability, and shape memory capabilities, which collectively aim to overcome the limitations of conventional aligner staging (5). Previous findings demonstrated that a single SMP aligner could potentially replace three sequential conventional aligners, offering advantages in terms of reduced treatment time, cost, plastic waste, and patient burden (5). While the results of this study are promising, several limitations must be acknowledged. The *in vitro* typodont model, which uses wax to simulate the periodontium, does not replicate the complex biological responses involved in bone remodelling during orthodontic tooth movement (19). Furthermore, the unique mechanical properties of SMPs differ significantly from traditional aligner materials (20, 21), limiting the generalizability of these findings. Future studies should focus on quantifying the forces generated by SMPs, evaluating their mechanical behaviour under clinical conditions, and comparing their efficacy with other aligner materials to validate their translational potential.

## Conclusion

The results of this study demonstrated that clear aligners fabricated from Graphy Tera Harz TC-85DAC resin possess the capability to induce mesial movement of the upper first molar. However, the type of tooth movement observed was limited to mesial tipping rather than true bodily movement. Across all study groups, the aligner designs resulted in consistent mesial tipping into the extraction space, indicating the current limitations of aligners in achieving controlled tooth movement in the mesial direction. These findings highlight the need for further investigations into alternative attachment designs or adjunctive biomechanical strategies using Finite Element Analysis to enhance root control and facilitate effective bodily mesialization using clear aligner therapy.

## Data Availability

The original contributions presented in the study are included in the article/Supplementary Material, further inquiries can be directed to the corresponding author.
